# Superior bioavailability of EPA and DHA from a L-lysine salt formulation: a randomized, three-way crossover study

**DOI:** 10.29219/fnr.v68.11028

**Published:** 2024-12-27

**Authors:** Christiane Schön, Antje Micka, Vishnupriya Gourineni, Roberta Bosi

**Affiliations:** 1 BioTeSys GmbH, Esslingen, Germany; 2 Evonik Corporation GmbH, Piscataway, NJ, USA; 3 Evonik Operations GmbH, Hanau-Wolfgang, Germany

**Keywords:** omega-3 fatty acids, eicosapentaenoic acid, docosahexaenoic acid, bioavailability, pharmacokinetic, lysine, ethyl ester, triglyceride

## Abstract

**Background:**

Omega-3 fatty acids, including eicosapentaenoic acid (EPA) and docosahexaenoic acid (DHA), are polyunsaturated fatty acids (PUFAs) with notable health benefits. Due to limited physiological production and insufficient dietary supply, external supplementation is important.

**Objective:**

This study aimed to compare the pharmacokinetics and bioavailability of EPA and DHA in AvailOm^®^ omega-3-lysine salt (Lys-FFA) versus standard ethyl ester (EE) and triglyceride (TG) formulations after a single oral dose in healthy subjects.

**Design:**

A randomized, three-way crossover study was conducted with 21 healthy subjects.

**Results:**

Twenty-one subjects (10 men, 11 women) completed the study. The average age was 41.7 years, and the mean body mass index was 23.0 kg/m^2^. The Lys-FFA formulation showed significantly higher uptake of omega-3 fatty acids (EPA+DHA combined and each individually) compared to EE. Specifically, Lys-FFA had a 9.33-fold (0–12 h) and 8.09-fold (0–24 h) higher bioavailability of EPA+DHA than EE and a 1.57-fold (0–12 h) and 1.44-fold (0–24 h) higher bioavailability than TG. ΔCmax and Tmax also favored Lys-FFA over EE.

**Discussion:**

Under fasting conditions, the absorption of EPA and DHA from EE is limited due to the need for enzymatic cleavage before absorption. This requirement is bypassed with Lys-FFA, which does not need cleavage.

**Conclusions:**

The study demonstrates that EPA and DHA lysine salt (Lys-FFA) offers superior bioavailability compared to EE and triglyceride forms, presenting a more effective supplementation option.

German Clinical Trials Register, DRKS-ID: DRKS00029183

## Popular scientific summary

- This study compares the pharmacokinetics and bioavailability of EPA and DHA in an omega-3-lysine salt versus standard ethyl ester and triglyceride formulations after a single oral dose in healthy subjects.
- Results demonstrate the superiority of supplementation with the lysine salt, thus offer a new effective option for supplementation due to its excellent bioavailability.
- Uptake profile of EPA and DHA differs, but superiority of the lysine salt could be confirmed for both fatty acids.

Omega-3-fatty acids are polyunsaturated fatty acids (PUFAs). The long-chain omega-3 fatty acids such as eicosapentaenoic acid (EPA) and docosahexanoic acid (DHA) have been known for various health benefits such as heart health and cognition (1–5). In general, omega-3 fatty acids play an important role in various physiological functions such as maintaining cell membrane integrity and acting as substrates for the production of prostaglandins and leukotrienes that mediate inflammatory responses (6, 7). The physiologic generation of long-chain omega-3 fatty acids from the short-chain omega-3 fatty acid alpha linolenic acid is limited (8). Thus, natural resources such as fish provide a source of omega-3 fatty acids. Additionally, a wide range of dietary supplements are available including alternative animal free (vegan) sources of omega-3 fatty acids produced from microalgae, which offer sustainable sourcing. Recently, there is increased demand for algae-sourced omega-3s globally (9). Omega-3 fatty acids are available as dietary supplements in the form of tablets, capsules, gummies, beverage drink-mix and melts (1–5). Typical supplements include formulations either in triglyceride or ethyl ester (EE) forms. However, these formulations need to be enzymatically cleaved prior to absorption, which limits the bioavailability depending on the external conditions, for example the fat content ingested along with the supplement (10, 11). To overcome this, and to enhance cellular absorption, an L-lysine salt formulation of carboxylic EPA and DHA was developed (10).

AvailOm^®^ omega-3 lysine salt consists solely of negatively charged, EPA- and DHA-rich free fatty acids as anions, which are combined with positively charged L-lysine as cation to form an omega-3 lysine salt in powder form. No other ingredients are used. Although the EPA- and DHA-rich fatty acids and lysine are combined in a 1:1 molecular ratio, their weight ratio is approximately 2:1 due to the different molecular weights of the fatty acid and lysine molecules. The product contains a minimum of 50% EPA and DHA and shows an excellent storage stability for active components as well as oxidation parameters. The free-fatty acid form does not require enzymatic cleavage or hydrolysis to be absorbed by cells. In a pilot study with eight young women, the improved bioavailability of the lysine salt formulation in comparison to EE form of omega-3 was already shown (10).

The objective of the present study was to compare the bioavailability of EPA and DHA of omega-3-lysine salt formulation with two different, conventional omega-3 fatty acid preparations such as EE and triglycerides in a healthy population of a larger sample size (*n* = 21) and of higher statistical relevance (in sex and age). Plasma levels of EPA and DHA were determined after a single oral dose of EPA and DHA delivered as omega-3-lysine salt in comparison to formulations of EPA and DHA in a standard EE or triglyceride formulation in healthy subjects.

## Present investigation

### Methods

#### Study design

The present study was performed as a controlled, randomized, and three-way cross-over study with three kinetic days with a 14-day wash-out in between. Subjects were randomly allocated to one of the three sequence groups according to randomization list. Study site was the Nutritional CRO BioTeSys GmbH in Esslingen, Germany. Subjects had to give informed consent before participating in the study. Prior to start, the study was approved by the Institutional Review Board (IRB) of Landesärztekammer Baden-Württemberg (Reference number: F-2021-069) and was conducted in accordance with the guidelines for Good Clinical Practice (GCP) set forth by the International Council for Harmonisation of Technical Requirements for Pharmaceuticals for Human Use (ICH) and in accordance with the Declaration of Helsinki regarding the treatment of human subjects in a study. Registration: German register DRKS00029183.

#### Subjects

**Fig. 1:**
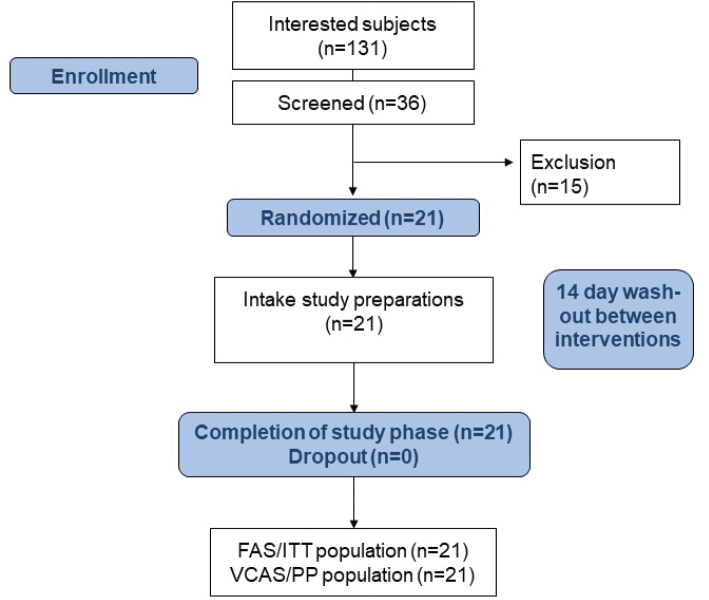
Study flow chart.

Between September 2022 and February 2023, 36 subjects were screened for their eligibility. 15 subjects resulted in screening failures, 21 subjects (11 women, 10 men) were included and completed the study (see Fig. 1). Main inclusion criteria were age 18 to 65 years, a body-mass index from 18.5 to 29.9 kg/m^2^, and a low fatty fish consumption defined as a frequency of less than twice per month. To exclude possible confounding factors, only subjects with a low to moderate omega-3-index (cut off level ≤ 6.0%) were included, and subjects were asked to refrain from fish consumption or other omega-3 fatty acid supplements or probiotics during the study intervention and to avoid plant oils rich in omega-3 fatty acids as well. Furthermore, subjects should keep their food and lifestyle habits.

### Study products

#### Test product

Investigational product was a preparation of a mixture of L-lysine salts of EPA and DHA-rich free fatty acids (abbreviated as Lys-FFA in the following), marketed as AvailOm^®^ 50 High EPA with a content of 31–37% EPA and 15–20% DHA (Evonik Operations GmbH, Essen, Germany). Free fatty acids for the production of Lys-FFA were derived from commercially available mixtures of EPA/DHA EEs.

#### Reference product 1

First reference product was a conventional fish oil supplement containing EPA and DHA in an approximately 1.5:1 ratio in EE form (abbreviated as EE in the following) from Nutravita Ltd, Berkshire, UK.

#### Reference product 2

Second reference product was a fish oil food supplement containing EPA and DHA in an approximately 1.5:1 ratio in triglyceride form (abbreviated as TG in the following) from Natural Elements GmbH Monheim am Rhein, Germany.

Products were applied as single dose under fasting conditions, each providing 1,100 mg EPA and DHA, which resulted in the intake of a different number of capsules depending on the study product (7 capsules of AvailOm^®^, 2 capsules of Nutravita, 3 capsules of Natural Elements).

### Blood sampling and assessments

Blood sampling at screening and each kinetic day was performed after at least 10 h overnight fast for the analysis of blood routine parameters including hemogram, lipid profile, hsCRP, creatine, and uric acid for kidney function and fasting glucose to confirm health status of the subjects. For efficacy parameters, the blood sampling was performed via permanent venous catheter for 12 h. After initial blood sampling for the assessment of baseline values, the study products were administered, and further samples were taken 0.75 h, 1.5 h, 2 h, 2.5 h, 3 h, 3.5 h, 4 h, 5 h, 6 h, 8 h as well as 12 h post dosing. Plasma of each time point was aliquoted and stored at -80°celsius until further analysis. In addition, omega-3 index was determined at screening and baseline assessments. Standardized meals were provided at the study day after 2 h (breakfast containing 18 g fat), 6 h (lunch), 9 h (snack) and 12 h (dinner).

For monitoring of safety concerns, tolerability and adverse events were asked at the end of each visit. Additionally, adverse events in the period between the two study days were recorded.

### Analysis of samples

Omega-3 index is the sum of EPA and DHA expressed as percentage of total fatty acids in the membrane of erythrocyte. Analysis was performed with gas chromatography (GC) at OmegaMetrix GmbH, Germany, to select the subjects at screening visit.

Determination of plasma fatty acids for efficacy parameters were performed at OmegaQuant, Sioux Falls, USA, by gas chromatography with flame ionization detection. Fatty acid composition was expressed as a percent of total identified fatty acids and concentrations as μg/mL of plasma. Routine parameters were determined at an accredited routine lab (Synlab Leinfelden-Echterdingen, Germany).

### Statistics

#### Sample size

The samples size was estimated based on the pilot study (10). A sample size of nine would provide approximately 80% power to detect at least a difference of 35% between the fatty acid forms by using a significance level of 5% in the cross-over design. To additionally allow stratified analysis by gender and considering a dropout rate and equally sized sequence groups, a total sample size of *n* = 21 was justified.

#### Analysis of pharmacokinetic parameters

Results have been analyzed using a linear mixed model taking into account sequence (three levels), period (three levels), product (three levels), and baseline omega-3 fatty acid within study period, as fixed effects, and subject as random effect (with different subject effects for each product, in order to take the unequal product variability into account and consequently fulfilling the normality assumption for the residuals). Due to the 14 days wash-out period, carry-over effects were not considered. Multiple pairwise comparisons of least-square means of iAUC were adjusted (by the method of Tukey) in order to assess differences between the lysine salt with the triglyceride and EE form.

Primary endpoint of the study was blood concentration of combination of EPA+DHA in plasma and determination of incremental area under the curve (iAUC) over a period of 12 h as well as comparison between the three study products. From concentration-time curves, pharmacokinetic endpoints iAUC, ΔC_max_, and T_max_ for two different time frames (0–12 h, 0–24 h) were calculated. iAUC was calculated applying the trapezoidal rule with y-axis defined by EPA+DHA value above baseline and the x-axis defined the sampling time points. In addition, the pharmacokinetic endpoints were determined for EPA and DHA separately. The pharmacokinetic endpoints iAUC and ΔC_max_ were analyzed after log transformation. Primary endpoint was evaluated for the entire population of *n* = 21 subjects. Just for exploratory motivation, an additional subgroup analysis by gender was performed for iAUC_0–12h_ (EPA+DHA).

**Fig. 2:**
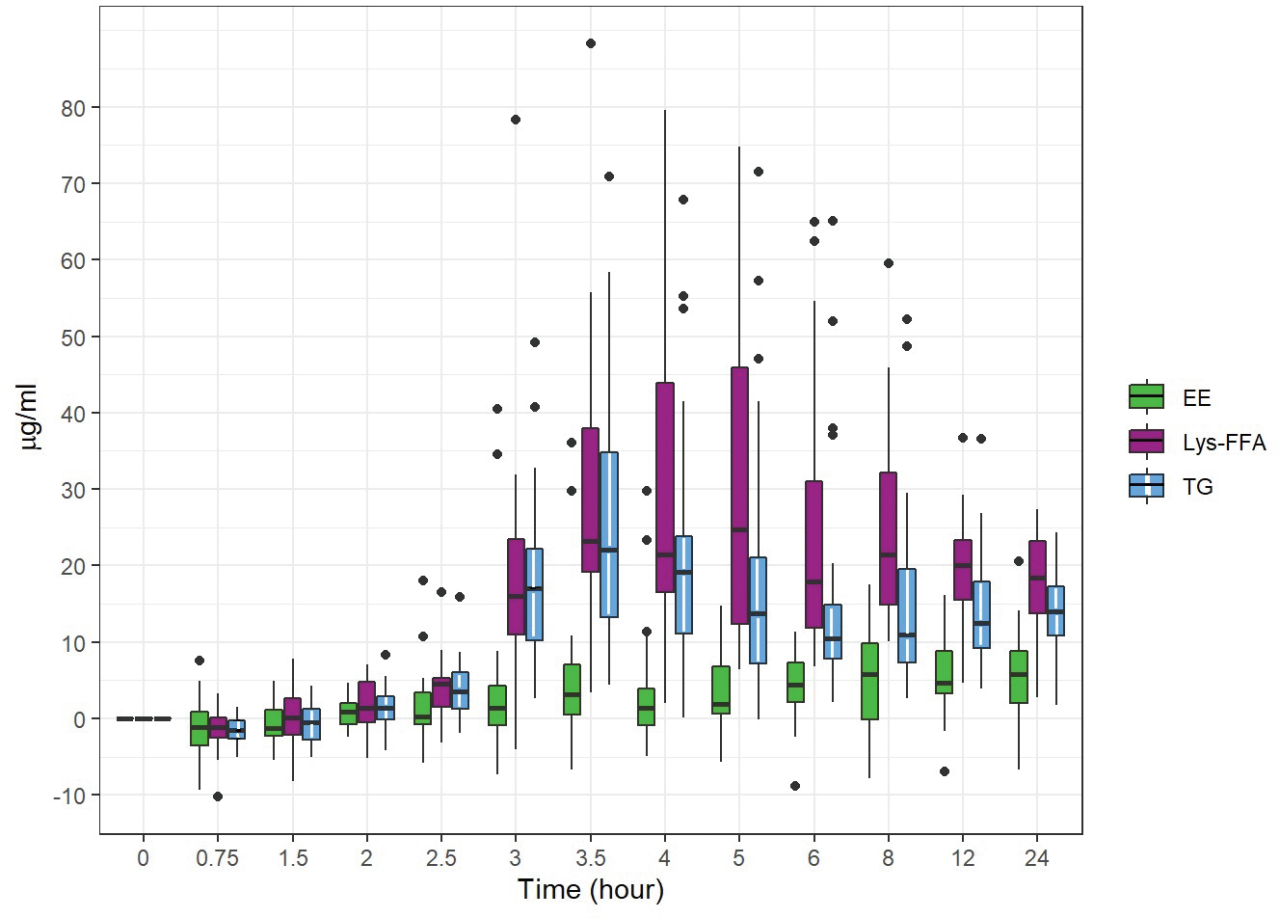
Baseline-adjusted and dose-adjusted EPA+DHA concentration obtained after the administration of the single dose of Lys-FFA versus EE and TG comparators (*n* = 21, each). Median, IQR, Whiskers: 1.5x IQR of EPA+DHA concentration in the plasma versus the 24-h time axis.

**Fig. 3:**
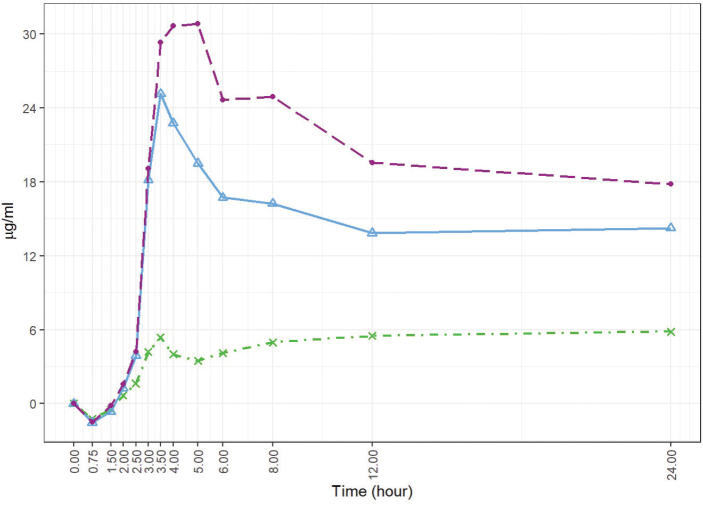
Baseline-adjusted and dose-adjusted EPA+DHA concentration in the plasma obtained after the administration of the single dose of Lys-FFA versus EE and TG comparators (*n* = 21, each). Arithmetic mean versus the 24-h time axis. • = *Lys-FFA (purple); Δ = TG (blue), x = EE (green)*

**Fig. 4:**
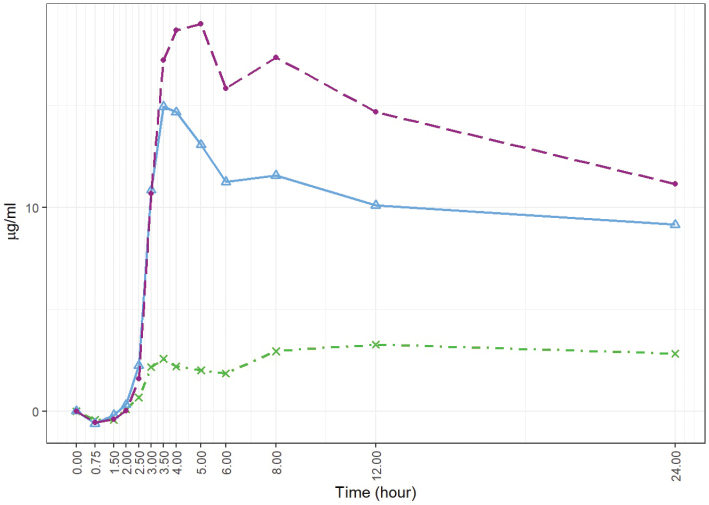
Baseline-adjusted and dose-adjusted EPA concentration obtained after the administration of the single dose of Lys-FFA versus EE and TG comparators (*n* = 21, each). Arithmetic mean of EPA concentration in the plasma versus the 24-h time axis. • = *Lys-FFA (purple); Δ = TG (blue), x = EE (green)*

**Fig. 5:**
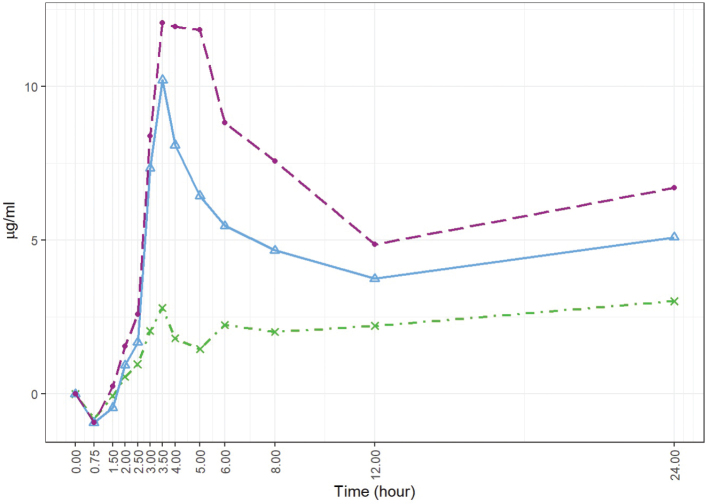
Baseline-adjusted and dose-adjusted DHA concentration obtained after the administration of the single dose of Lys-FFA versus EE and TG comparators (*n* = 21, each). Arithmetic mean of DHA concentration in the plasma versus the 24-h time axis. • = *Lys-FFA (purple); Δ = TG (blue), x =EE (green)*

Figure 2 displays the boxplots of the baseline-adjusted plasma EPA+DHA concentrations. Figure 3 displays the arithmetic mean value of EPA+DHA in plasma for all subjects at each time point (Figs. 4 and 5 refer to EPA and DHA, respectively).

As commercial products were used, the EPA+DHA concentration was matched best possible; however, especially the ratio of EPA to DHA differed between products. Therefore, to allow a direct comparison, the pharmacokinetic endpoints were evaluated after dose normalization.

## Results

### Study collective

Twenty-one subjects (10 men and 11 women) were enrolled in this study between October 2022 and February 2023. There were no dropouts, and all subjects concluded the study according to the protocol. Characteristics of study participants are summarized in Table 1. As study products were administered under supervision of study site staff, compliance was 100%. Omega-3 index was assessed at baseline at each kinetic day indicating equal starting conditions.

**Table 1:** Baseline characteristics of study collective

Variables	Study Population Mean (± 95% CI)
Age [years]	41.7 (95% CI: 35.9–47.5)
Body-mass-index [kg/m^2^]	23.0 (95% CI: 21.9–24.1)
Omega-3-index at screening	4.7 (95% CI: 4.3–5.1).
Systolic blood pressure (mmHg)	124.2 (95% CI: 115.0–133.4)
Diastolic blood pressure (mmHg)	79.4 (95% CI: 73.7–85.2)
Total cholesterol (mg/dl)	203.0 (95% CI:187.7–218.4)

### Efficacy parameters

#### Primary endpoint iAUC0–12h for EPA + DHA

**Fig. 6:**
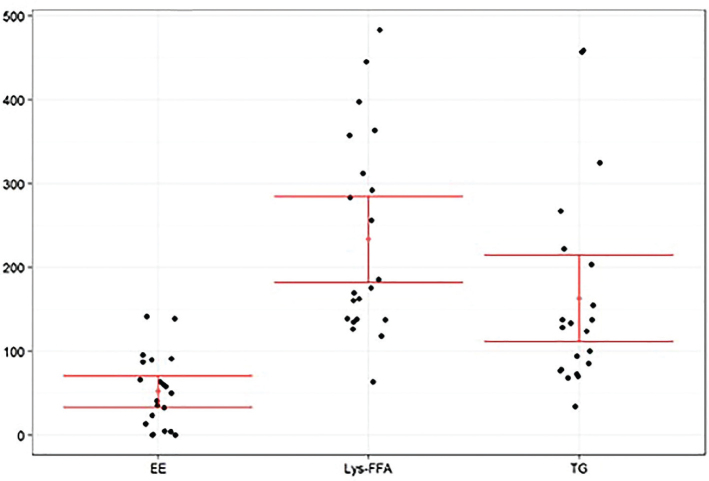
Distribution of iAUC_0–12h_ of EPA+DHA dose adjusted [μg/mL*h]. A Scatter diagram with mean ± 95 % CI for the *n* = 21 values is shown for EE, Lys-FFA and TG.

iAUC_0–12h_ demonstrated on average highest concentrations after intake of Lys-FFA formulation followed by TG and lowest concentrations after EE application. At time 0.75 h, all three treatments showed decreasing values with respect to the baseline and then rise again after 1.5 h. The plasma concentrations were highly significant increased over time starting from 3 to 24 h (*P* < 0.001 in repeated measures ANOVA and Dunnett’s test) after product intake in comparison to the baseline values for Lys-FFA and TG. For EE, the increase was less pronounced with only a significant difference versus baseline at 3.5 h and 8 to 24 h (see Figs. 2 and 3). In terms of relative bioavailability (as measured by the geometric LS-mean for iAUC_0–12h_), Lys-FFA resulted in approximately 9.33 times the value for total EPA+DHA compared to EE and 1.57 times the value for total EPA+DHA compared to TG (see Table 3). Least square means are shown in Table 2. For completeness, we mention that TG had 5.92-fold higher availability of EPA+DHA against EE. The magnitude of the spread can be better appreciated by considering the 90% confidence interval. All results are statistically significant and show that Lys-FFA is not bioequivalent to the other treatments (since the confidence interval is higher than 1.25). In order to visualize the spread difference, Fig. 6 displays the scatterplot of the measured PK parameter iAUC_012h_ of EPA+DHA. Here Lys-FFA has the highest values and variability. Also, TG has high variability, while EE has concentrated low values.

**Table 2:** Estimated least square means and 95% confidence interval for PK parameters iAUC_0–12h_, iAUC_0–24h,_ ΔCmax_0–12h_, ΔCmax_0–24h_ for EPA+DHA dose adjusted, grouped by treatment

EPA+DHA	Lys-FFA	EE	TG
	LS mean (95% CI)	LS mean (95% CI)	LS mean (95% CI)
iAUC_0–12h_	204.38 (157.59, 267.74)	21.98 (9.21, 52.46)	130.32 (92.76, 183.09)
iAUC_0–24h_	424.11 (347.23, 518.01)	52.46 (19.49, 139.77)	295.89 (232.76, 372.41)
ΔC_max, 0–12h_	32.79 (24.78, 42.95)	5.93 (3.22, 10.91)	25.28 (18.36, 34.81)
ΔC_max, 0–24h_	33.45 (25.53, 43.38)	6.69 (3.56, 12.43)	26.31 (19.69, 35.16)

**Table 3:** Estimated ratio of geometric least-square means and 90% confidence interval for PK parameters iAUC_0–12h_, iAUC_0–24h_, ΔCmax_0–12h_, ΔCmax_0–24h_ for EPA+DHA dose adjusted. The ratios are shown corresponding to the comparisons.

EPA+DHA	Lys-FFA vs EE	*P*	Lys-FFA vs TG	*P*
	Geo LS mean ratio (90% CI)		Geo LS mean ratio (90% CI)	
iAUC_0–12h_	9.33 (4.12, 21.12)	<0.0001	1.57 (1.25, 1.99)	0.0005
iAUC_0–24h_	8.09 (3.06, 21.41)	0.0002	1.44 (1.15, 1.79)	0.0039
ΔC_max, 0–12h_	5.5 (3.26, 9.28)	<0.0001	1.29 (0.98, 1.71)	0.1387
ΔC_max, 0–24h_	4.99 (2.85, 8.71)	<0.0001	1.27 (0.99, 1.62)	0.1198

The higher bioavailability of Lys-FFA was also confirmed for the observation time frame of 0 to 24 h with 8.09-fold higher availability of EPA+DHA in comparison to the EE supplement and a 1.44-fold higher availability than the TG supplement. Similar results could also be observed for EPA and DHA alone (see Figs. 4 and 5). For both outcomes, there was a significant difference between Lys-FFA and the EE supplement, whereas greater differences were observed for EPA (iAUC_0–12h_ ratio of geometric LS-means 25.54-fold) in comparison to DHA (iAUC_0–12h_ ratio of geometric LS-means 4.69-fold).

### ΔC_max_ for EPA + DHA

For the pharmacokinetic parameter ΔC_max_ for the time interval 0–12 h, a significantly higher availability of Lys-FFA could be shown compared to the EE supplement (geometric LS-mean of the Lys-FFA is 5.50 times higher). Both products are therefore not considered as bioequivalent. Geometric LS-mean of Lys-FFA is 1.29 times higher than the triglyceride product, however, not statistically significant. Regarding ΔC_max_ for the interval 0–24 h, Lys-FFA resulted in a higher bioavailability for EPA+DHA (4.99-fold for EE and 1.27-fold TG, without statistical significance for TG; see Tables 2 and 3).

### T_max_ for EPA+DHA

The uptake of EPA+DHA was significantly faster after intake of Lys-FFA in comparison to EE. This was confirmed by a paired Wilcoxon test for both observation periods 0–12 h (*P* = 0.0181) and 0–24 h (*P*-0.0047; see Table 4). The faster uptake of Lys-FFA was also confirmed for EPA and DHA separately. However, a difference in the time to reach C_max_ between Lys-FFA and TG could not be shown (see Table 4).

**Table 4:** Tmax values of the plasma profile of EPA+DHA: median and IQR range of the PK parameters Tmax_0–12h_ and Tmax_0–24h_ of Lys-FFA, EE and TG. Wilcoxon test is used for comparison.

EPA + DHA	Lys-FFA	EE	TG	Lys-FFA vs EE	Lys-FFA vs TG
	Median (Q1, Q3)	Median (Q1, Q3)	Median (Q1, Q3)	Wilcoxon test P	Wilcoxon test P
T_max, 0–12h_	5 (3.5, 6)	8 (5.5, 12)	3.5 (3.5, 5)	0.0181	0.4202
T_max, 0–24h_	5 (3.5, 6)	8 (6, 24)	3.5 (3.5, 8)	0.0047	0.9433

## Safety and tolerability

### Tolerability

Subjects rated overall tolerability as ‘well tolerated’, ‘slightly unpleasant’, ‘very unpleasant’, and commented on possibly occurring side effects like fishy aftertaste, burping, or nausea. Tolerability was assessed after 24 h after the blood sampling on the kinetic days. Overall tolerability was good. 14 out of 21 rated Lys-FFA as ‘well-tolerated’. Six subjects rated it as ‘slightly unpleasant’ and one subject as ‘very unpleasant’ due to fishy aftertaste and burping. For the reference product in triglyceride form, two subjects mentioned a ‘slightly unpleasant’ tolerability after intake. Overall, tolerability of the EE form was very good.

#### Adverse events

During kinetic days in total 10 adverse events were reported. Mainly (6x) headache was reported presumably linked to the avoidance of caffeine and the long sitting period. Other adverse events were cystitis, vertigo, allergic reaction to the patch of the cannula, and gastrointestinal pain. No interference of these adverse events was expected on outcome parameters, and none of these AEs was related to study products. Additional adverse events reported during wash-out phases were not in conflict with continued study participation and recovered at study days. The results of this study did not raise any safety concerns.

## Discussion

Omega-3 fatty acids (namely EPA and DHA) intake – either via the diet or by the use of supplements/functional food products – is associated with several potential health benefits, such as a reduced risk for CVD or lower triglyceride levels. In addition to the intake via fatty fish such as salmon or herring, dietary supplements are very popular as the supply from the diet remains often below the recommendations (12). Given the low dietary intakes of EPA and DHA in Europe and USA, EPA and DHA dietary supplements are options to help fill the gap between typical and recommended intakes (13). A wide range of dietary supplements are available for those wanting to increase their intakes (6). This study focused on three omega-3 forms with different biochemical structures.

Results from the present study showed a significantly higher uptake of omega-3 fatty acids (sum of EPA+DHA) after administration of Lys-FFA in comparison to products containing the EE or triglyceride form. However, it can be claimed that both Lys-FFA and the triglyceride product have a higher availability than the EE product. This underlines the hypothesis that the EE form requires emulsification via bile salts and hydrolysis by pancreatic lipases, which is why this formulation requires the co-ingestion of fat (10). Other previous studies also demonstrated that EPA and DHA bound as EE are only absorbed to a small extent when not consumed with a fat-containing meal (14). In contrast to this, the underlying principle of the new omega-3 Lys-FFA formulation is the dissociation of the salt in the acidic environment of the stomach, allowing the free fatty acids to be absorbed from the small intestinal tract without prior hydrolysis by lipases. This means that the combined intake of fat, necessary to induce the secretion of pancreatic lipases, is not required for absorption of EPA and DHA from the lysine salt formulation. In a previous study, the lysine salt was only compared to an EE form (10); therefore the focus of the present study was particularly on the comparison of bioavailability between Lys-FFA and TG. Meaningful differences have been demonstrated in the present study for the iAUC_0–12h_ and for the combination of EPA+DHA for Lys-FFA compared to TG. This emphasizes the outstanding absorption profile of the novel formulation. TG is rapidly metabolized in the intestine by pancreatic lipase (15), whereas Lys-FFA is already dissociated in the stomach and absorbed in the small intestine. Interestingly, the speed of uptake of TG was slightly faster than that of Lys-FFA, without significant difference. One thing to bear in mind here is that products can have different delivery formats (e.g. soft gel or hard capsule). This may also explain the minimal side effects that occurred in this study, and further intake recommendations should be adapted to this. However, it is very likely that the side effects experienced by subjects are also due to taking the product on an empty stomach.

In this study, 10 male subjects and 11 female subjects were involved. Although significant difference between male and female could not be shown in this study, other studies indicated that sex hormones could have an influence on fatty acid metabolism (16). With respect to the small sample size, we conclude that the topic of gender difference should be further investigated and clarified only in presence of a sample size much larger than in this study.

## Conclusion

In summary, the study demonstrates the superiority of a supplementation of EPA and DHA in the form of a lysine salt in comparison to other commonly marketed omega-3-fatty acids forms like EEs or triglycerides and thus offers a new effective option for supplementation due to its excellent bioavailability.
